# The amyloid imaging for the prevention of Alzheimer's disease consortium: A European collaboration with global impact

**DOI:** 10.3389/fneur.2022.1063598

**Published:** 2023-01-20

**Authors:** Lyduine E. Collij, Gill Farrar, David Valléz García, Ilona Bader, Mahnaz Shekari, Luigi Lorenzini, Hugh Pemberton, Daniele Altomare, Sandra Pla, Mery Loor, Pawel Markiewicz, Maqsood Yaqub, Christopher Buckley, Giovanni B. Frisoni, Agneta Nordberg, Pierre Payoux, Andrew Stephens, Rossella Gismondi, Pieter Jelle Visser, Lisa Ford, Mark Schmidt, Cindy Birck, Jean Georges, Anja Mett, Zuzana Walker, Mercé Boada, Alexander Drzezga, Rik Vandenberghe, Bernard Hanseeuw, Frank Jessen, Michael Schöll, Craig Ritchie, Isadora Lopes Alves, Juan Domingo Gispert, Frederik Barkhof

**Affiliations:** ^1^Department of Radiology and Nuclear Medicine, Amsterdam University Medical Center, location VUmc, Amsterdam, Netherlands; ^2^Amsterdam Neuroscience, Brain Imaging, Amsterdam, Netherlands; ^3^GE Healthcare, Amersham, United Kingdom; ^4^Barcelona Beta Brain Research Center, Barcelona, Spain; ^5^Centre for Medical Image Computing, and Queen Square Institute of Neurology, UCL, London, United Kingdom; ^6^Laboratory of Neuroimaging of Aging (LANVIE), Université de Genève, Geneva, Switzerland; ^7^Synapse Research Management Partners, Barcelona, Spain; ^8^Department of Neurobiology, Care Sciences and Society, Center of Alzheimer Research, Karolinska Institutet, Stockholm, Sweden; ^9^Department of Nuclear Medicine, Centre Hospitalier Universitaire de Toulouse, Toulouse, France; ^10^Life Molecular Imaging GmbH, Berlin, Baden-Württemberg, Germany; ^11^Janssen Pharmaceutica NV, Beerse, Belgium; ^12^Alzheimer Europe, Luxembourg, Luxembourg; ^13^Ace Alzheimer Center Barcelona, Universitat Internacional de Catalunya, Barcelona, Spain; ^14^Networking Research Center on Neurodegenerative Diseases (CIBERNED), Instituto de Salud Carlos III, Madrid, Spain; ^15^Department of Psychiatry, University Hospital of Cologne, Cologne, North Rhine-Westphalia, Germany; ^16^Faculty of Medicine, University Hospitals Leuven, Leuven, Brussels, Belgium; ^17^Institute of Neuroscience (IONS), Université Catholique de Louvain, Brussels, Belgium; ^18^Department of Psychiatry and Neurochemistry, University of Gothenburg, Gothenburg, Sweden; ^19^Centre for Clinical Brain Sciences, University of Edinburgh, Edinburgh, Scotland, United Kingdom; ^20^Brain Research Center, Amsterdam, Netherlands

**Keywords:** amyloid, positron emission tomography (PET), consortium, Alzheimer's disease, diagnosis, prognosis

## Abstract

**Background:**

Amyloid-β (Aβ) accumulation is considered the earliest pathological change in Alzheimer's disease (AD). The Amyloid Imaging to Prevent Alzheimer's Disease (AMYPAD) consortium is a collaborative European framework across European Federation of Pharmaceutical Industries Associations (EFPIA), academic, and ‘Small and Medium-sized enterprises’ (SME) partners aiming to provide evidence on the clinical utility and cost-effectiveness of Positron Emission Tomography (PET) imaging in diagnostic work-up of AD and to support clinical trial design by developing optimal quantitative methodology in an early AD population.

**The AMYPAD studies:**

In the Diagnostic and Patient Management Study (DPMS), 844 participants from eight centres across three clinical subgroups (245 subjective cognitive decline, 342 mild cognitive impairment, and 258 dementia) were included. The Prognostic and Natural History Study (PNHS) recruited pre-dementia subjects across 11 European parent cohorts (PCs). Approximately 1600 unique subjects with historical and prospective data were collected within this study. PET acquisition with [^18^F]flutemetamol or [^18^F]florbetaben radiotracers was performed and quantified using the Centiloid (CL) method.

**Results:**

AMYPAD has significantly contributed to the AD field by furthering our understanding of amyloid deposition in the brain and the optimal methodology to measure this process. Main contributions so far include the validation of the dual-time window acquisition protocol to derive the fully quantitative non-displaceable binding potential (BP_*ND*_), assess the value of this metric in the context of clinical trials, improve PET-sensitivity to emerging Aβ burden and utilize its available regional information, establish the quantitative accuracy of the Centiloid method across tracers and support implementation of quantitative amyloid-PET measures in the clinical routine.

**Future steps:**

The AMYPAD consortium has succeeded in recruiting and following a large number of prospective subjects and setting up a collaborative framework to integrate data across European PCs. Efforts are currently ongoing in collaboration with ARIDHIA and ADDI to harmonize, integrate, and curate all available clinical data from the PNHS PCs, which will become openly accessible to the wider scientific community.

## 1. The scientific landscape of Alzheimer's disease

Dementia is a major cause of disability, dependency, and mortality in the elderly population. It is estimated that by the year 2050, up to 150 million individuals will be affected by this condition (1). Care of these patients comes with considerable societal and economic impact, stressing the importance of optimal diagnostics and the availability of disease-modifying therapies. The main cause of dementia is Alzheimer's disease (AD), which is a neurodegenerative disorder that progressively impairs cognitive functioning (primarily memory and executive functioning). One of the first observable changes in the AD brain is the accumulation of the amyloid-β (Aβ) protein, which can be detected *in vivo* by positron emission tomography using radiolabeled tracers. Currently, three fluorine-18 radiotracers have been approved for clinical use by the European Medicine Agency (EMA) and by other competent authorities worldwide; [^18^F]flutemetamol/Vizamyl^TM^ (FMM) (2) by GE Healthcare, [^18^F]florbetaben/Neuraceq^TM^ (FBB) (3) by Life Molecular Imaging, and [^18^F]florbetapir/Amyvid^TM^ (FBP) (4) by Eli Lilly. The detection of amyloid pathology supports a clinical diagnosis of AD and provides useful information on its clinical progression (5). After some years of clinical use of the amyloid PET tracers, the appropriate use criteria (AUC) were drafted (5, 6) and today amyloid-PET imaging is more frequently used in a clinical setting. However, reimbursement of the technique is lagging due to the lack of definitive evidence supporting its clinical utility and cost-effectiveness in the diagnostic workup.

In the clinical trial setting, the role of amyloid-PET has increased significantly over the past decade. Initial trials did not require biomarker confirmation at study entry, and amyloid-PET was therefore rarely used as an inclusion criteria, resulting in a high fraction of enrolled subjects being amyloid-negative (6, 7). As the field advances, biomarker confirmation for trial inclusion has become the standard and nowadays amyloid-PET is generally used as a quantitative measure of amyloid burden for both trial enrollment and to assess target engagement. As both ongoing and future trials are moving from an interventional to a preventive approach, the role of amyloid-PET imaging in clinical trial design is again changing. Also, the arrival of plasma biomarkers, which are being actively developed at present, will most likely have an important role in future clinical and research settings (8) and already have a prominent role in screening participants for trial enrollment (9), challenging the use of amyloid-PET imaging. Nonetheless, the technique holds the advantage of being the only validated measure against neuropathology as the gold standard (10), in contrast to fluid biomarkers, PET provides regional information and a measure of the extent of Aβ pathology (11), and is able to support disease monitoring efforts (12).

## 2. The innovative medicines initiative ‘AMYPAD’ study

It is within this context, that the Innovative Medicines Initiative (IMI) funded the ‘Amyloid Imaging to Prevent Alzheimer's Disease’ (AMYPAD) study. Since its original kick-off in October 2016, the AMYPAD consortium is a unique collaboration of a wide range of partners, including nine academic institutes, three industry/ European Federation of Pharmaceutical Industries Associations (EFPIA) (GE Healthcare [GEHC], Life Molecular Imaging [LMI] and Janssen Pharmaceuticals), 2 ‘Small and Medium-sized enterprises’ (SME's) (IXICO and SYNAPSE), and 1 patient organization (Alzheimer Europe) (www.amypad.eu). With the funding formally ended in at the end of September 2022, AMYPAD has formed new collaborations with ARIDHIA and Alzheimer's disease Data Initiative (ADDI) to maintain, curate, and provide access to the large database of biomarkers collected from nearly 2400 subjects who have been included in the study at large. In this paper, we want to highlight the important collaborations necessary to make AMYPAD a successful project, reach not only the scientific community but also engage society at large, and illustrate how these endeavors ensured the value of the consortium during and after the funding period.

## 3. The AMYPAD studies

The AMYPAD consortium is led by Professor Frederik Barkhof from the Amsterdam UMC, location VUmc, and Dr. Gill Farrar from GE Healthcare. AMYPAD aimed to optimize the use of amyloid-PET in both clinical and research settings. Also, collaborators had a strong desire to develop a robust analytical methodology to ensure that measures of the amyloid burden by PET are both accurate and consistent across different centres and multiple tracers. To these ends, two trials were set-up: the *Diagnostic and Patient Management study (DPMS)* including a memory clinic population; and the *Prognostic and Natural History Study (PNHS)*, focused on a pre-dementia and mainly pre-clinical population.

### 3.1. Diagnostic and patient management study (DPMS)

AMYPAD DPMS aimed to assess the clinical impact and cost-effectiveness of amyloid-PET in memory clinic patients. One of the AMYPAD DPMS main strengths is its randomized controlled study design. Participants were allocated to three study arms: ARM1, amyloid-PET performed early in the diagnostic workup (within 1 month); ARM2, late in the diagnostic workup (after 8 ± 2 months); or ARM3, if and when the managing physician chose to scan the subject. This allowed comparing a diagnostic pathway that includes amyloid-PET (ARM1) with one without amyloid-PET (ARM2). The study recruitment was finalized in October 2020 and a total of 840 participants with variable cognitive stages (244 with subjective cognitive decline plus [SCD+], 341 with mild cognitive impairment [MCI], and 255 with dementia) were enrolled from eight memory clinics, resulting in the largest European study implementing amyloid-PET in clinical practice. The main outcome was the difference between ARM1 and ARM2 in the proportion of participants receiving an etiological diagnosis with very high diagnostic confidence after 3 months. As a secondary outcome, we are assessing the cost-effectiveness of amyloid-PET by using longitudinal health-related outcomes and information on the participants' use of healthcare resources. Please refer to Frisoni et al. (13) and (14) for a detailed description of the study rationale and baseline features of the final recruited patient population, respectively.

### 3.2 The prognostic natural history study (PNHS)

**Figure 1:**
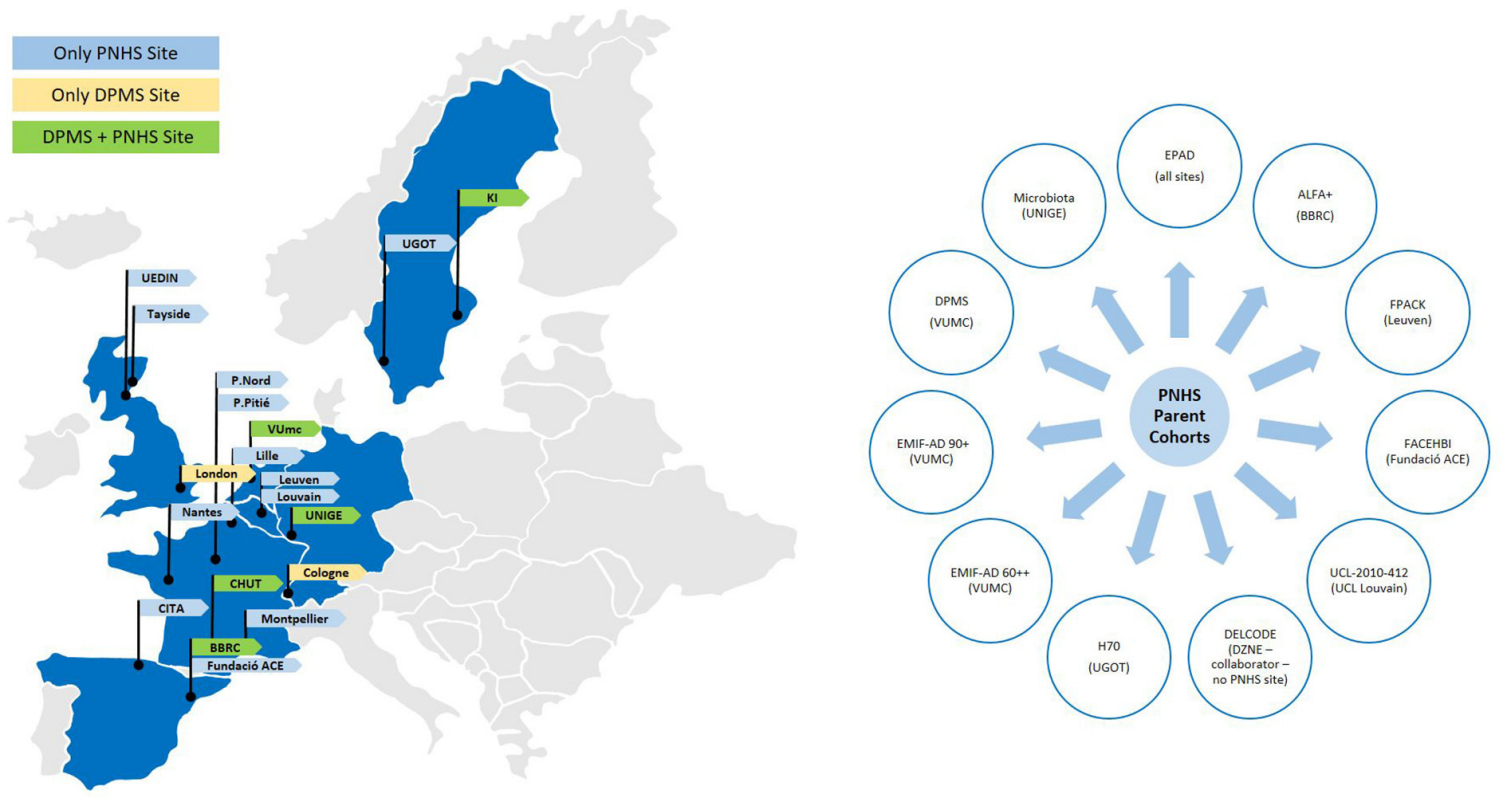
Network of study cohorts and sites that contribute to the AMYPAD consortium. Sites affiliated with the DPMS study are shown in yellow, sites affiliated with the PNHS are shown in blue, and sites affiliated with both trials are shown in green. *EPAD*: European Prevention of Alzheimer's Dementia; *ALFA*+ for Alzheimer and Families; *FPACK* Flemish Prevent AD Cohort-KU; *FACEBHI* Fundació ACE Healthy Brain Initiative; *UCL-2010-412* University College Louvain 2010-412 study; *DELCODE* Longitudinale Studie zu Kognitiven Beeinträchtigungen und Demenz; *H70* Gothenburg H70 Birth cohort study; *EMIF-AD 60*++ *and 90*+ Medicine Initiative European Medical Information Framework for AD Twin60++ and 90+ study from the Alzheimercenter Amsterdam; *DPMS* Diagnostic and Patient Management Study from AMYPAD.

Originally, the PNHS was closely associated with its sister project ‘European Prevention of Alzheimer's dementia’ (EPAD), aiming to perform amyloid-PET in this well-phenotyped cohort to investigate the added value of this imaging technique in assessing a participant's risk to develop cognitive decline due to AD. However, to facilitate timely recruitment into the study, other cohorts with similar aims and readily collected data across Europe were invited to participate as parent cohorts (PCs). In return, AMYPAD PNHS provided the newly collaborating PCs with the opportunity to perform amyloid-PET imaging. Effectively, this framework boosted the recruitment for PNHS and resulted in the availability of longitudinal data in a significant proportion of participants in several studies across Europe. To date, 17 centres have contributed to the PNHS across 11 PCs [EPAD (15), EMIF-AD 60++ (16) and 90+, ALFA+ (17), FACEHBI (18), FPACK (19), UCL-2010-412, Microbiota, AMYPAD DPMS [*via* the VUmc] (13), DELCODE (20), and H70 (21)], with several additional PCs expressing interest in joining forces after the IMI-funding period. By the end of the study in June 2022, 1,192 prospective baseline and 227 follow-up scans had been performed. An additional 1,300 PET scans were also made available through collaborations with the PCs, bringing the final total available scans for PNHS analysis to over 2,700 PET images across 1,624 participants. Please see Lopes Alves et al. (22) for an overview of the study design and scientific aims of the PNHS. An overview of AMYPAD affiliated sites can be found in Figure 1.

## 4. The value of EFPIA partnerships

### 4.1. Availability of PET radiotracers

Beyond the academic collaborations, a key partnership within AMYPAD was the support of our EFPIA partners through the supply of the EMA-approved [^18^F]flutemetamol (FMM) and [^18^F]florbetaben (FBB) PET radiotracers by GE Healthcare (GE) and Life Molecular Imaging (LMI), respectively. Both GE and LMI maintain distribution networks in Europe to provide respective tracers to investigators; imaging sites in the AMYPAD consortium were chosen so that there was a relatively equal distribution of manufacturing availability of the two PET tracers between the AMYPAD study centres. The short shelf life of these F-18 radiolabeled tracers (~8–10 h) limits the geographic distribution of the products and therefore careful logistical planning between manufacturing sites, nuclear medicine departments, and referring physicians was required to optimize the utility of each batch produced. A working party was specifically set up for the duration of clinical scanning to pay careful attention to the consistent delivery of both tracers to facilitate including the maximum numbers of subjects for both DPMS and PNHS.

As per standard guidelines, 185MBq (FMM) or 300MBq (FBB) of tracer were injected intravenously and 20-min scans were acquired 9- min post-injection. All PNHS images were centrally collected by IXICO and processed using their in-house LEAP pipeline (23), providing global and regional Centiloid (CL) values. For the DPMS, amyloid-PET scans were processed and analyzed using AMYPYPE, a modified Cortex ID (24) PET-only pipeline, which provides global CL units as well as regional z-scores compared to a reference population. For 515 PNHS participants, dynamic amyloid-PET scans were performed with the so-called coffee-break protocol (25), which allows for full quantitation (i.e., BP_*ND*_) and additionally provides a measure of relative flow (i.e., R_1_), in addition to CL values. In addition, 318 of these participants had, at least, one longitudinal dynamic amyloid-PET scan. Dynamic amyloid-PET was performed longitudinally in a sub-set of DPMS (*n* = 45), bringing the total number of collected ‘coffee-break’ scans over 900 and making AMYPAD a unique resource to study in what scenarios dynamic amyloid PET imaging could be advantageous over standard acquisition and quantification.

### 4.2. Regulatory interactions

One of the fundamental premises of IMI partnerships is to ensure that the technology that is widely used in research can also optimally be used for routine clinical workup. Both PET tracers used in the AMYPAD consortium (FMM and FBB) were previously approved by the EMA through pivotal phase III registration studies wherein a high correlation was verified between visual inspection of the images, as either negative or positive, for neuritic amyloid and the *post-mortem* measures of amyloid burden. However, further studies relating to the value of the amyloid-PET agent to improve diagnostic thinking were suggested by EMA, and hence the DPMS was designed also to investigate this component. In fact, dialogue with both EMA and Health Technology Assessment instances (HTAs) was first conducted in 2016, with a goal to incorporate EMA's input into the study design *via* formal Scientific Advice, as well as initiating dialogue with HTA bodies. A second Scientific advice was conducted in 2019, providing further input, particularly in the area of quantitative methodology for measuring amyloid load using PET. Specifically, focus was given to EMA's view on the opportunity for quantitative metrics, such as the Centiloid measure, to assist with both subject selection and therapy monitoring, as well as for prediction of cognitive progression and measuring small early changes over time. During this period, quantitation was added to the Summary of Product Characteristics (SmPCs) of both tracers used in AMYPAD, as a result of data packages presented to EMA showing the value of quantitation as an adjunct to the visual read of a adjunctive diagnostic scan.

AMYPAD aims to continue discussions with various regulators, such as EMA and FDA, even beyond its IMI period, to facilitate a wider appreciation of both the robustness and value of quantitative methodology to measure amyloid PET burden.

### 4.3. Interacting with other IMI partners and external collaborators

AMYPAD had a close working relationship with EPAD (https://ep-ad.org/) in that a large number of the initial PNHS participants were recruited from the Longitudinal Cohort Study (LCS). EPAD has also developed data access models that AMYPAD benefited from (see section 8 below). Additionally, AMYPAD has been an active member of coordination and support action (CSA) NEURONET (https://www.imi-neuronet.org/), which was created to set up an efficient platform to boost synergy and collaboration across IMI's wider neurodegenerative disorders (ND) portfolio. Here, members of AMYPAD were represented on the NEURONET Scientific Coordination Board, the Working Group on sustainability and the NEURO Cohort Task Force. Furthermore, AMYPAD's cohorts, datasets and algorithms were signposted in the NEURONET Asset Map. This in turn was held on the NEURONET Knowledge Base, which also signposts and reports further information about the AMYPAD project, including its deliverables, partners and publications (https://kb.imi-neuronet.org/). Other close relationships have developed with other global consortiums. Collaborations with IDEAS, ALFA, AIBL and ADNI are ongoing, whilst data sharing with additional cohorts such as OASIS, EMIF-AD, ABIDE, and ADC yielded the highly cited work on the pooled multi-tracer amyloid staging model (26) (see section 6.1).

## 5. Amyloid burden in Centiloid units for DPMS and PNHS

The goals of AMYPAD rely on the assumption that amyloid burden can be accurately quantified irrespective of the radiotracer that was used for the acquisition of the PET scans. In this regard, the Centiloid (CL) method has been proposed as an absolute scale to quantify amyloid burden, allowing the pooling and comparison of data across tracers and quantification pipelines. This scale assigns a CL value of 0 to the lack of amyloid burden (similar to what would be observed in a young control group), and a CL value of 100 to the typical amyloid load of mild-moderate AD patients.

To verify the assumption that CL values are comparable across the two tracers used in AMYPAD, we have conducted a Gaussian Mixture Modeling (GMM) exercise on the distribution of CL values in the DPMS and the PNHS. GMM is a data-driven statistical technique capable of estimating the parameters of a finite number of Gaussian distributions that underlie the observed distribution of values. GMM has been widely used to model global estimates of amyloid burden as measured by PET (27). It is well-established in the literature that the distributions of amyloid load values, when recruiting memory clinic patients, show a bimodal distribution with one Gaussian modeling the distribution of ‘negative’ scans and another one that of the ‘positive’ ones (28, 29). Such a bimodal distribution fits well with the clinical use of the amyloid tracers that are typically rated visually as positive or negative, but it is not suitable to describe the distribution observed in cognitively unimpaired individuals at high risk of AD, which is dominated by a Gaussian centred around zero CL that is skewed toward higher values (27). Such a distribution violates the assumption of the GMM that the data points follow a finite number of Gaussian distributions. In addition, GMM presents other limitations such as sensitivity to the initialization parameters, and the lack of spread estimates (i.e., the 95% confidence interval [95%CI]) of the estimated parameters (relative proportion, mean and standard deviation).

To overcome such limitations and robustly model the distribution of CL values also in this early population, we have introduced several methodological innovations to the modeling. First, to circumvent the dependency of initial estimates and the lack of spread estimates of the Gaussian parameters, we have implemented a bootstrapped version of the GMM. This method performs a GMM with random initial parameters in 100,000 bootstrap samples from the original distribution. Bootstrapping is a technique that randomly resamples a given distribution with replacements for a high number of times. By doing so, it mimics the sampling of the recruited population in the study and is, therefore, capable of providing generalizable estimates. Using this method, we can also obtain spread estimates to the Gaussian parameters and, due to the random initialization at each of the bootstrap samples, we compensate for the dependency of the GMM to the initial parameters

**Figure 2:**
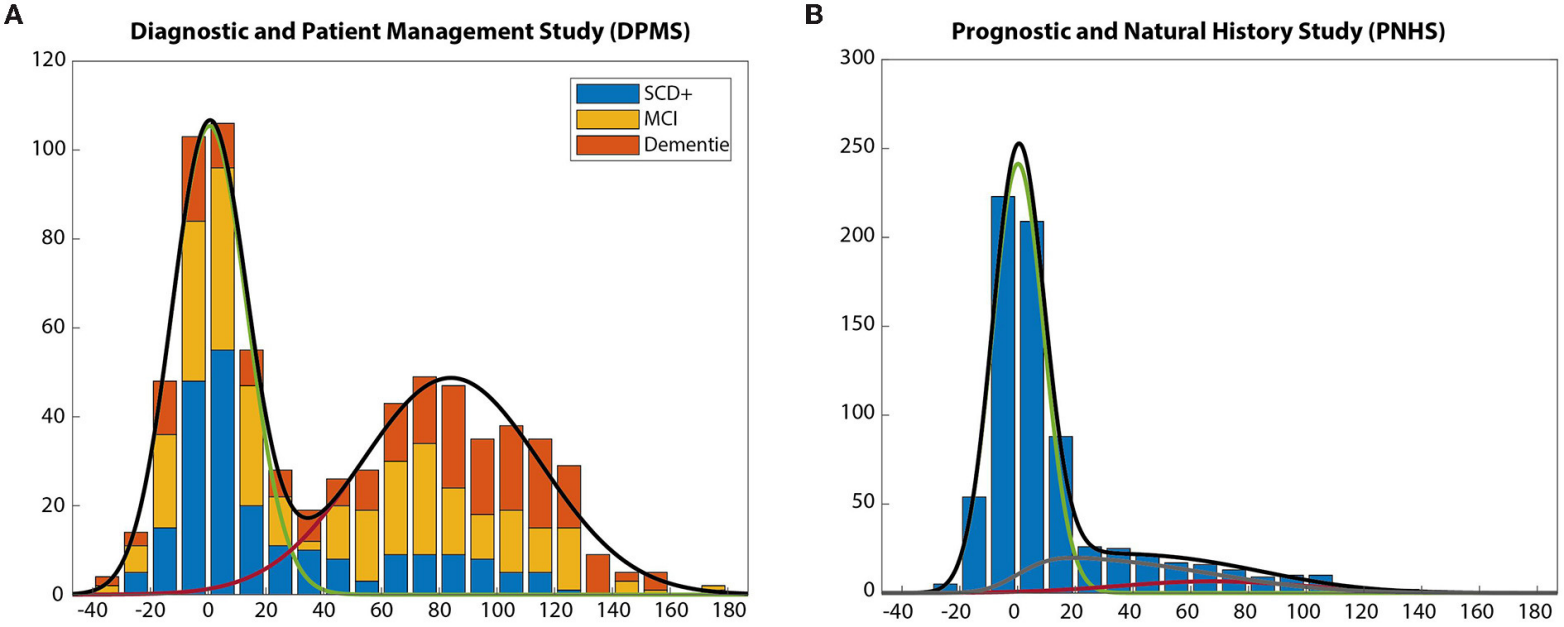
Centiloid distributions across DPMS and populations. **(A)** Centiloid distribution across patient populations, reflecting a bi-modal distribution. **(B)** Centiloid distribution across per-dementia subjects, mostly cognitively unimpaired, skewed toward lower amyloid burden.

Finally, to overcome the limitation of some of the distributions not resulting from a finite number of “pure” Gaussian distributions, a non-Gaussian distribution has been added to the GMM to model the intermediate CL values. The distribution of these intermediate CL values is modeled using a dedicated function that is linked to the means and standard deviations of the positive and negative Gaussians and only the relative proportion of intermediate values is estimated by the GMM. This strategy is based on previous work on the modeling partial volume voxels of magnetic resonance scans (30). Using this procedure, we modeled the distribution of the CL values in the DPMS study, also stratifying it by tracer (Figure 2A).

Of note, our version of the GMM estimated a negative Gaussian with a mean of 0.42 CL, close to zero as expected, with 95% CI below 2 CL [−0.94, 1.90]. The mean of the positive Gaussian was 92.52 CL, slightly below the value of 100 CL expected for a group of typical mild-moderate AD patients. Since the DPMS included amyloid-positive participants at earlier AD clinical stages (SCD+, MCI) such a lower CL value was also expected. Moreover, when stratifying by tracer, the 95%CI of all parameters overlapped between the two tracers, thus confirming that the CL scale provides comparable estimates of amyloid burden across the two tracers in the DPMS.

Regarding the PNHS, the developed GMM method could also adapt to the expected distribution that was dominated by a negative Gaussian skewed toward lower values. In this case, it can be observed that the relative proportion of the distribution of intermediate values is higher (20%) than that of the positive one (7%). In this case, the 95%CI of the mean value of the negative Gaussian also included the zero, as expected (Figure 2B).

## 6. Pan-European scientific collaborations

In addition to scientific data generated from both the DPMS and PNHS, AMYPAD researchers have significantly contributed to the AD field by furthering our understanding of amyloid deposition in the brain and the optimal methodology to measure this process. Several key papers have been published using either locally readily available datasets, open-access sources, or the academic collaborations established under the AMYPAD umbrella.

From a methodological perspective, the consortium has validated the implementation of the coffee-break or dual-time acquisition protocol for both the FMM and FBB radiotracers (25). This acquisition protocol results in fully quantitative data (i.e., BP_*ND*_) and additionally provides a surrogate measure of cerebral blood flow (i.e., R_1_) while allowing for interleaved scanning, which translates to efficient scanner use and reduced participant burden (9). Subsequently, we investigated the value of fully quantitative measures in the context of clinical trials, showing that sample sizes in AD secondary prevention trials can be reduced by the acquisition of dynamic PET scans and/or by restricting inclusion to subjects with intermediate amyloid burden or *APOE*-ε4 carriers. Moreover, using a targeted early composite leads to reductions in sample size requirements in primary prevention trials (31).

The concept of an early composite is the focus of a second major line of research within the AMYPAD consortium, namely the value of regional rather than global amyloid-PET investigations to improve disease tracking, risk profiling, and prediction of cognitive decline over time. A major collaboration was the development of a multi-tracer staging model, which included over 3,000 amyloid-PET scans from six cohorts, including historical data of several PCs aligned with the PNHS (26). Taking these findings, we performed preliminary analyses in predicting changes in cognitive functioning in a preclinical population of the OASIS-3 open-access dataset. We showed that regional and longitudinal amyloid-PET improved the prediction of cognitive decline in specific domains (mean follow-up period was 4.0 ± 1.9 years) (32). This is considered the groundwork for the primary end-point of the PNHS trial.

The third line of research has been to optimize the use of amyloid-PET in the clinical setting. Firstly, from a regional perspective, implementing the results of the previously mentioned quantitative studies in our approach to performing visual assessments. We showed in a collaborative paper between VUmc and BBRC that visual assessment of amyloid-PET images can identify early amyloid accumulation and grade the extent of deposition. This approach goes beyond the use of a binary global measure, currently implemented in the clinical routine. Moreover, our results were confirmed by *post-mortem* data from the Phase III Flutemetamol trial, kindly provided by GEHC (33).

Our most recent focus is on the implementation of (Centiloid) quantification into the clinical routine, to not only support visual assessment of challenging cases, but also prepare the field for a potential necessity which could arise from the possible approval of disease-modifying therapies in the near future. To this end, academic and EFPIA partners collaborated on a comprehensive review regarding possible quantification approaches for the clinical setting (34) as well as engaging with regulatory bodies to share the in-depth knowledge that AMYPAD has gained using these methods during the course of the project. In this context, the AMYPAD team has investigated the robustness of the Centiloid quantification method (35), its feasibility in detecting early Aβ pathology (36), and its ability to detect changes over time. This work has been collated into a Biomarker Qualification Opinion document, which has been submitted to the EMA at the end of September 2022.

## 7. AMYPAD success beyond the trials: SYNAPSE and Alzheimer Europe

Management, communication, and dissemination were a core part of the AMYPAD project to ensure that activities and results have been communicated and shared with internal and external stakeholders in a clear, consistent, and effective manner. To combine an adequate use of resources and a successful outreach, Synapse Research Management Partners (SYNAPSE) and Alzheimer Europe work in close collaboration with all project partners.

Firstly, a Project Management Office was set up to follow up on project activities and to monitor compliance with the work plan, planned resources and schedule according to IMI2 JU rules. SYNAPSE, a firm specialized in the high-quality management of complex research and development projects in the biomedical sector, led the management activities of the project including areas such as financial management (e.g., monitoring budget and resource consumption), legal (e.g., amendments or subcontracting of study centers), risk management, and deliverable quality control procedures. The day-to-day management was crucial for the completion of the deliverables and the achievement of project milestones, and the establishment of the project governance facilitated the collaboration with other related initiatives (including EPAD, NEURONET, European Platform for Neurodegenerative Diseases [EPND], and ADDI).

Secondly, a communication plan for the AMYPAD project, led by Alzheimer Europe, was developed at an early stage of the project. In this plan, a consistent communication strategy was defined, to provide continuous up-to-date information about the project and disseminate its results among different stakeholders, but also to liaise and establish synergies with neighboring initiatives. This strategy was adopted throughout the project execution and targeted a variety of key audiences, including among others the patient community, regulators, payers, policymakers, and the wider scientific community. Specific attention was paid to reaching out to the dementia community. Alzheimer Europe used its extensive network of 37 member associations from 33 countries and its communication tools (e.g., website, social media, newsletter, Dementia in Europe magazine, annual conference) to relay information on the AMYPAD project. This represented a major opportunity to target Alzheimer's associations/patient groups affiliated with Alzheimer Europe. In addition, AMYPAD communication objectives were met thanks to tailored strategies and the use of cross-channel communication. AMYPAD communication tools such as the project's website (https://amypad.eu/) and the active presence on social media channels such as Twitter maximized the outreach by creating continuous visibility of the project and engagement with stakeholders in the discussion on the different topic areas covered by the project.

## 8. Toward open source

### 8.1. Imaging harmonization

#### 8.1.1. PET harmonization

**Figure 3:**
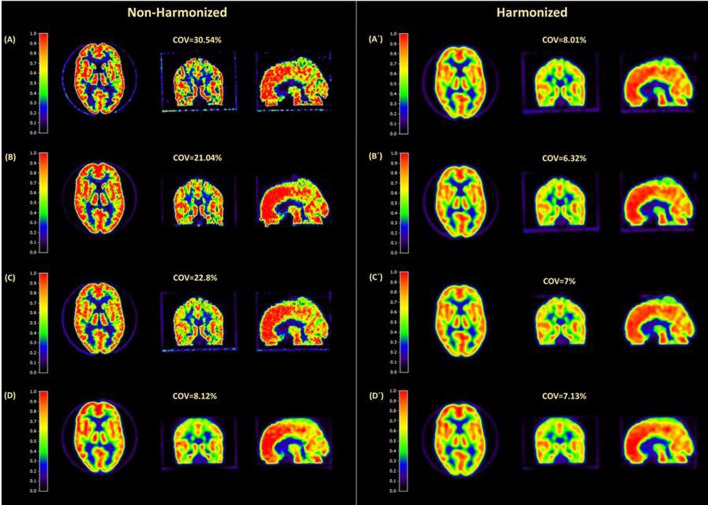
Visual illustration of amyloid PET harmonization results. Example images of the Hoffman phantom were acquired on four different scanners before **(left panel)** and after **(right panel)** harmonization. Coefficient of variance (COV%), which is an indication of image noise, is shown for each scan. Before harmonization, the COV% difference was more than 22, while after harmonization this ranged only ~1.

It is well established that the Centiloid scale, used in the two trials in AMYPAD, is robust to differences in image resolution and quality (35). Given such a robust behavior, it could have been argued that there was no need to harmonize the differences in image resolution and quality inherent to multi-center PET studies. However, this may not hold true when estimating regional amyloid burden, as opposed to global ones. In this regard, one of the goals of AMYPAD is to better understand the information provided by regional patterns of amyloid deposition, on top of global estimates as the CL scale. As an example of such added value, we recently described three distinct spatial-temporal trajectories of amyloid accumulation (37) and proposed a visual staging method based on the regional pattern of positivity of the PET scans (33). Since AMYPAD will serve to assess the clinical value of the regional information of PET scans, an image harmonization standard operational procedure (SOP) has been developed in collaboration with EARL (https://earl.eanm.org/), the initiative of the European Association of Nuclear Medicine (EANM), to harmonize quantification in nuclear medicine imaging. This SOP is based on the acquisition of Hoffman phantom scans in the AMYPAD imaging network to account for inter-scanner differences and provide several indicators of image quality (Figure 3) (38).

#### 8.1.2. Advanced MRI harmonization

**Figure 4:**
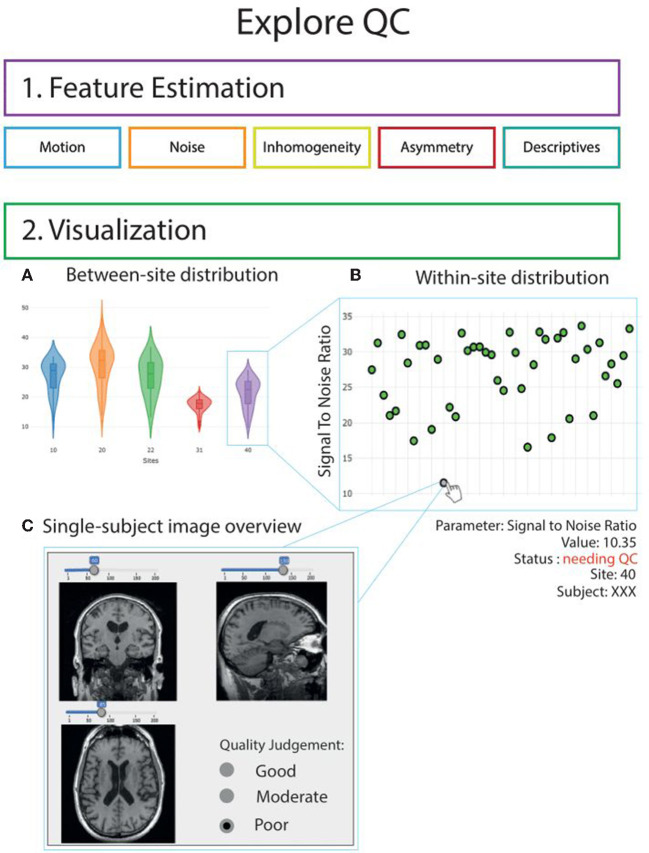
Graphic illustration of ExploreQC toolbox. Overview of the quality control workflow. QC features are computed in the feature estimation module and cover 5 image features domains. Feature distributions can then be interactively inspected between-sites (5A) and within-sites (5B). Single-subject scans can be opened by clicking on the scatterplots (5C). Adapted from Lorenzini et al. (39).

In addition, most PNHS PCs had available historical advanced magnetic resonance imaging (MRI). These include resting-state functional MRI (rs-fMRI), diffusion-weighted imaging (DWI) and arterial spin labeling (ASL). The PNHS team has, therefore, aligned efforts with the ‘EPAD imaging core’ to process all collected scans using a harmonized pipeline as described in Lorenzini et al. (39). To promote accessibility and replicability, standard image-derived phenotypes (IDPs) will be computed from MRI sequences and shared as spreadsheets. IDPs are image-specific summary statistics that provide a quantitative way to investigate structural and functional brain characteristics. For rs-fMRI, a group-level independent component analysis (ICA) will be performed on 4 mm MNI-registered bold time series, using FSL Melodic (40) to identify canonical resting-state networks (RSN). A dual regression approach will then be used to compute the mean time series and functional connectivity strength of each RSN. Similarly, bold time series will be summarized within atlases region of interest. Functional connectivity matrices in atlas space and graph properties will be derived. For DWI, pre-processed data will first be fed into the FSL Brain Extraction Toolbox (BET) (41) and then into FSL DTIFIT, to fit the diffusion tensor model to the data and produce diffusion tensor imaging (DTI) scalars maps (fractional anisotropy (FA), and mean (MD), axial (AD) and radial (RD) diffusivity). On these data, the Tract-based spatial statistics (TBSS) pipeline will be used to compute global and regional FA features from the JHU ICBM-DTI-81 atlas (42). For ASL, mean cerebral blood flow (CBF) and spatial coefficient-of-variation will be computed as described in Mutsaerts et al. (43). These processing steps have been integrated in an in-house workflow to perform semi-automatic QC of MRI data. This set of QC functionalities was written as an extension to ExploreASL (43) called ExploreQC (39). The semi-automated QC procedure was based on two steps: feature estimation and visualization. Image quality features were computed from five image feature domains: motion, noise, inhomogeneity, asymmetry, and descriptives. The visualization module consists of an interactive dashboard with violin and scatter plots for observing variation between and within sites, respectively. Individual scans can be visually inspected by selecting their data points on the scatter plots, allowing to visualize the scans themselves together with the QC features (Figure 4). The toolbox in freely available online (https://github.com/luislorenzini/ExploreQC).

Overview of the quality control workflow. QC features are computed in the feature estimation module and cover 5 image features domains. Feature distributions can then be interactively inspected between-sites (5A) and within-sites (5B). Single-subject scans can be opened by clicking on the scatterplots (5C). Adapted from Lorenzini et al. (39).

### 8.2. Clinical data harmonization

Different strategies were used for the harmonization of the clinical data in the AMYPAD trials, mostly determined by the design of each study. The prospective nature of the DPMS allowed for the implementation of harmonized strategies already from the beginning of the project, which were executed during the whole data collections. However, the PNHS dataset is composed by the combination of prospective and historical data from multiple sources, these limited the capacity to define harmonize methods during data collection, as most data was already obtained, and required a more thoroughly process of harmonization across the different data sources.

The AMYPAD DPMS clinical dataset includes baseline and follow-up variables concerning sociodemographic, clinical, and cognitive features of 840 memory clinic patients. These data were prospectively collected locally by the teams of the 8 AMYPAD DPMS recruiting memory clinics using electronic case report forms (developed by IXICO) and, therefore, following harmonized procedures defined in advance during the early phases of the study. Then, after data collection, the AMYPAD DPMS dataset had a final quality-checked by the sponsor team (University of Geneva).

Meanwhile, the AMYPAD PNHS clinical dataset is a combination of prospective and historical data from 17 European sites. Due to the variety of sources and data formats present across the Parent Cohorts, the data curation process in PNHS deals with multiple challenges. Among these obstacles, the most notables are the use of different data models, measurements, and cognitive questionnaires. Therefore, it was decided to perform a comprehensive process of data curation based on the work of the Data Curation Network (https://datacurationnetwork.org) which developed a standardized set of CURATED steps (Check, Understand, Request, Augment, Transform, Evaluate, and Document).

This process resulted in the largest European dataset phenotyping longitudinally individuals at risk of AD-related progression, which currently consists of ~3,350 subjects, ~1,600 of those with a baseline amyloid PET and about 940 of them having at least one follow-up PET acquisition. The dataset currently contains 9,740 observations (visits) and 614 variables, grouped into (68) “concepts” and (13) “domains,” such as demographics, family history, genetics, vital signs, medical history, neuropsychological questionnaires, lifestyle, CSF, PET and MRI. While current dataset has been developed using its own data model, tailored to the needs of the project, the AMYPAD PNHS has been selected to work with the European Health Data & Evidence Network (EHDEN) in the adoption of the OMOP data model. This will allow for the systematic analysis of the PNHS database, using a harmonize format as well as a common presentation of terminologies, vocabularies and coding schemes (EHDEN has received funding from the IMI 2 Join Undertaking under the grant agreement No 806968).

All this process of data handling has been performed in close collaboration with the ARIDHIA team, where their expertise in data science has played a major role supporting data integration, harmonization and storage.

### 8.3. Availability of software

A couple of open-source software packages dedicated to PET imaging in dementia have been developed: NiftyPET for neuro-image reconstruction with basic analyses, and NiftyPAD for dynamic PET analyses.

**Figure 5:**
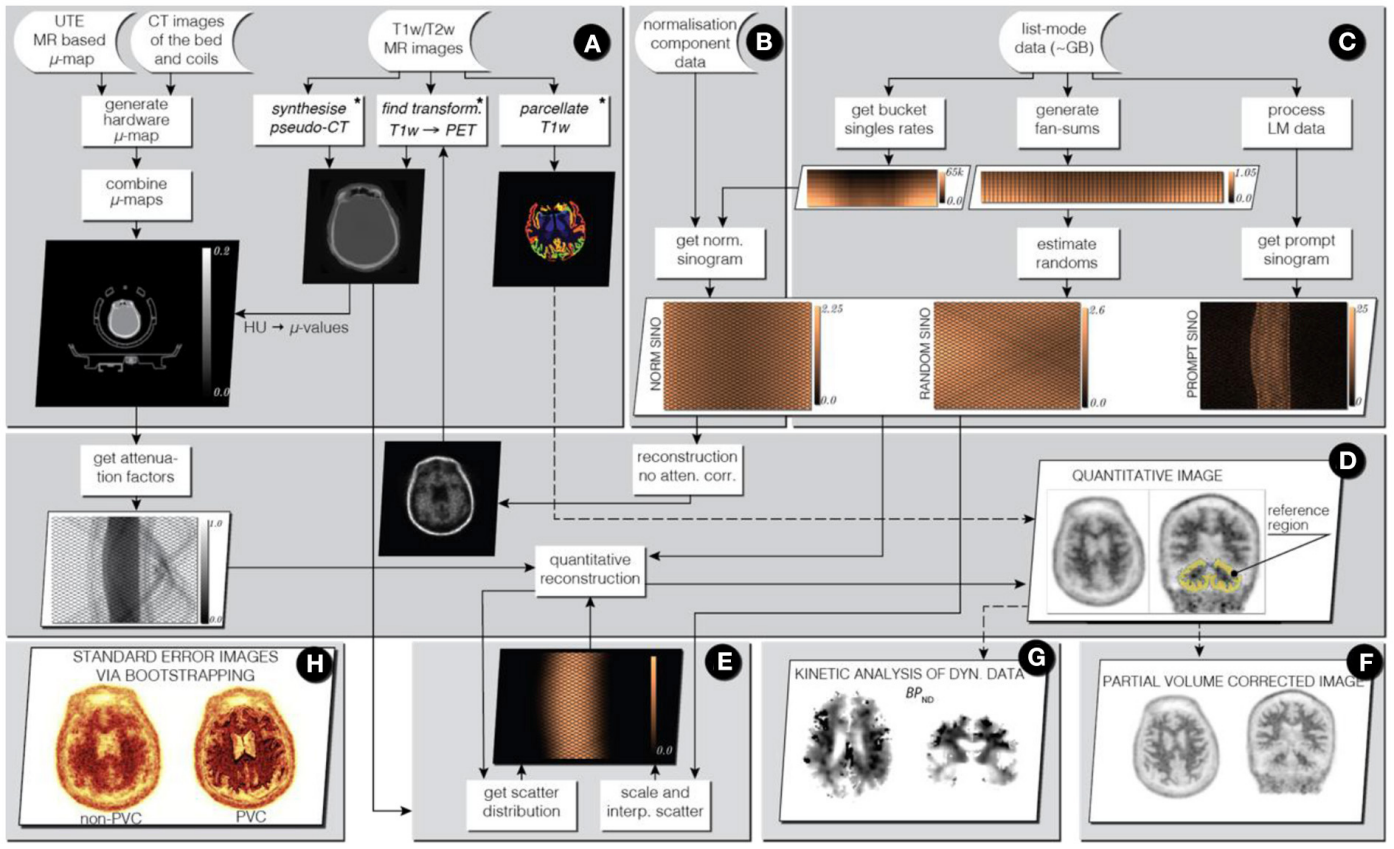
Infrastructure for standalone PET image reconstruction and analysis of PET/MR brain data using amyloid PET tracer. Stages **(A–C)** involve processing of input data (raw acquisition and image data), while in stages **(D, E)** image reconstruction is performed followed by image analysis in stages **(F–H)**.

*NiftyPET* (https://niftypet.readthedocs.io/) is an open-source software solution for standalone and high-throughput PET image reconstruction, manipulation, processing and analysis with high quantitative accuracy and precision (Figure 5) (44). One of its key applications is brain imaging in dementia using amyloid and tau tracers. The key computational routines are written in CUDA C for fast and efficient execution on NVIDIA GPU devices. The routines are then embedded in Python C extensions to be readily available for high-level manipulation of PET data in Python. Using *NiftyPET*, it has been possible to accurately assess the precision of MR-PET image registration, critical for accurate quantification of amyloid PET data (45). Also, the software was used for comprehensive analysis of the American College of Radiology PET phantom to estimate the spatial resolution of PET scanners (46) – information which is essential for performing a robust partial volume correction of amyloid PET images.

*NiftyPAD* is a freely available open source, Python-based software package for versatile analyses of static, full or dual-time window dynamic brain PET data. The key novelties of NiftyPAD are the analyses of dual-time window scans with reference input processing, pharmacokinetic modeling with shortened PET acquisitions through the incorporation of arterial spin labeling (ASL)-derived relative perfusion measures, as well as optional PET data-based motion correction. The implemented kinetic models were validated by comparing the outcomes with the well-established software packages PPET and/or QModeling. Real dynamic PET data were used from four different amyloid tracers used in clinics. High correlations were earlier validated software indicating reliable model implementation in NiftyPAD. It is freely available (https://github.com/JJiao/NiftyPAD), and allows for multiplatform usage. The modular setup makes adding new functionalities easy, and the package is lightweight with minimal dependencies, making it easy to use and integrate into existing processing pipelines.

### 8.4. Facilitating an open-access platform

#### 8.4.1. Data access

**Figure 6:**
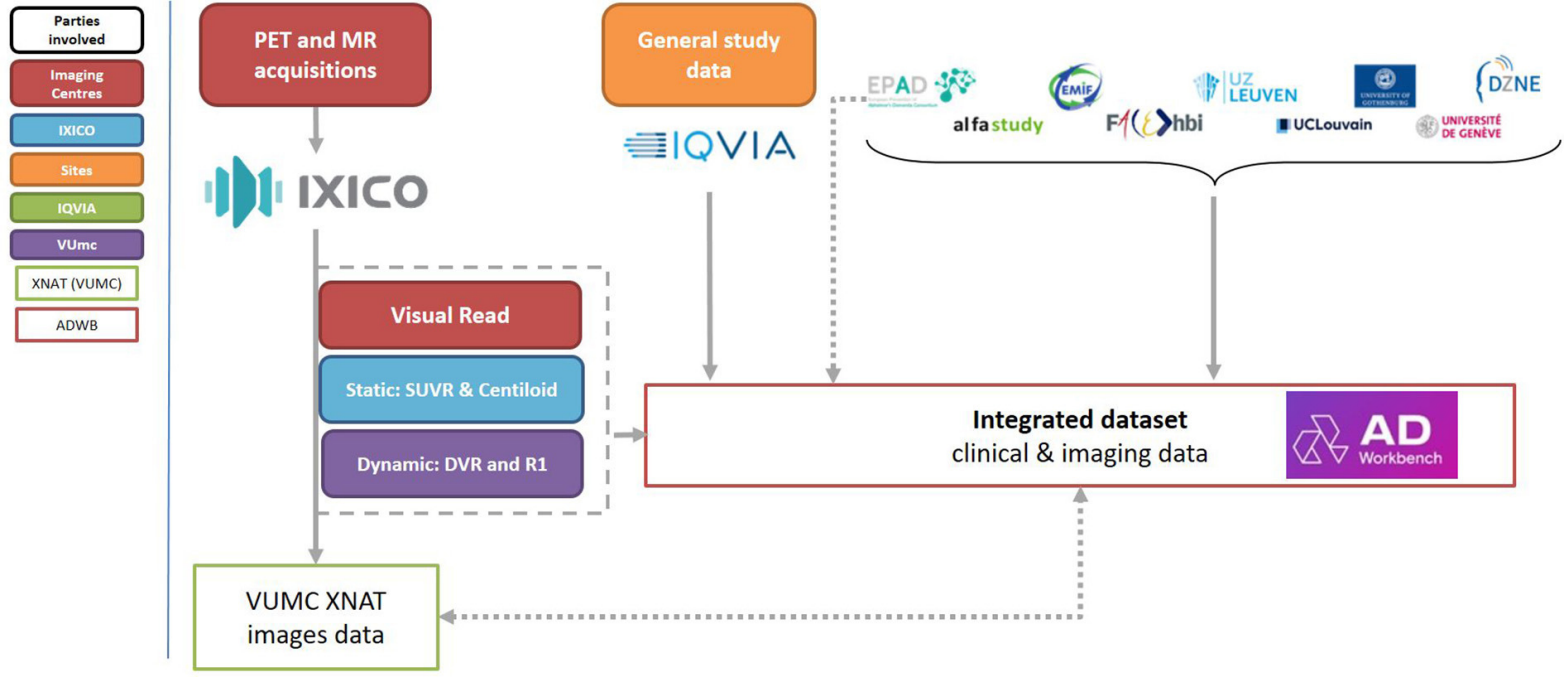
Schematic representation of data-flow within the PNHS trial.

The AMYPAD PNHS dataset is hosted in the Alzheimer's Disease Data Initiative (ADDI) Workbench, with the first private data release made in November 2021 (Figure 6). Thanks to a 5-year partnership between the AMYPAD consortium and ADDI, the PNHS dataset will remain available to the research community beyond the project duration, with the first public release planned by the end of the first quarter of 2023.

Those researchers interested in using the AMYPAD PNHS data can request access to the imaging, clinical, and biomarker data for scientific research investigation and/or educational activities. The application can be performed *via* the FAIR Data Service of the Alzheimer's Disease Data Initiative (ADDI). In this platform, the user will indicate if the request includes only access to the clinical data or also to the neuroimaging data, the data domains, and the type of data (i.e., raw, harmonized, or derivative). In addition, the researcher should provide a one-page proposal describing the study and the use of the data.

The AMYPAD Data Sharing and Publication Committee (DPC) will review the application and the research proposal. Incomplete applications or those without a clear focus will not receive approval. The results of the DPC review will be sent *via* the FAIR platform, and approved application will be processed differently based on the requested data type:

- *Harmonized and derivative data* does not require further approval by the Parent Cohorts, and the access will be granted. This process will take up to 1 month.
- *Raw data* requires specific approval by the Parent Cohorts, which will be contacted with a copy of the proposal. Each cohort will decided if they would grant or not approval. This process will take up to 2 months (1 month for the assessment of the DPC and 1 month for the Parent Cohort).

The results for the data access request will be sent to the researcher *via* the FAIR platform, and approved application will receive access to retrieve the data in the AD Workbench. In case that neuroimaging data was also requested, information to access the XNAT will be also provided *via* the FAIR platform (more details in the next section).

#### 8.4.2. Image data access and XNAT

Imaging data from all sites have been collected by IXICO and have undergone quality control and between-site harmonization. Image data are disseminated by the Amsterdam UMC using an XNAT system (www.xnat.org), an open-source medical image server that allows control of multi-user access and storage of clinical non-imaging data. The image data that is made available, adheres to the PET-BIDS standard (https://bids-specification.readthedocs.io) (47), which ensures transparency of the image provenance and processing history, and enables open and reproducible science.

Together with the EPAD project, the AMYPAD group is working in conjunction with the Alzheimers Disease Data Initiative (https://www.alzheimersdata.org), which will ensure the availability of the main clinical databases for these projects, and support sharing of the imaging data as facilitated by Amsterdam UMC.

## 9. Conclusion

In summary, the AMYPAD consortium has made a strong contribution to the AD field over the last 6 years. A legacy of over 3,500 amyloid PET scans covering the entire AD *continuum* has been collected across the DPMS and PNHS, which is now curated for sharing with the research community. AMYPAD has expanded the knowledge in both the utility and measurement of amyloid PET beyond the basic dichotomization of a standard negative or positive scan and, in particular, has harnessed the Centiloid metric as a universal tracer-independent method for assessing amyloid load. The consortium has widely demonstrated the robustness and validity of the technique across tracers to enable further research using this technology for both initial diagnosis and prognosis, and opens possibilities for optimal therapy monitoring and/or patient-management.

## Data availability statement

The data supporting the conclusions of this article will be made available by the authors, without undue reservation.

## Ethics statement

The studies involving human participants were reviewed and approved by Medical Ethical Committee of the University Medical Center Amsterdam, location VUmc and all local sites. The patients/participants provided their written informed consent to participate in this study.

## Author contributions

All authors listed have made a substantial, direct, and intellectual contribution to the work and approved it for publication.
